# Nitrous Oxide as an Anæsthetic

**Published:** 1872-08

**Authors:** H. Endermann


### ARTICLE IV.

### *Nitrous Oxide as an Anæsthetic.*

BY H. ENDERMANN, PH. D.

An unfortunate occurrence, which happened a few weeks ago, and which resulted in the death of a lady, has caused a good deal of comment in our daily and weekly papers, criticising the preparation and application of nitrous oxide. I have been enabled to not only examine the methods of preparation and application, but have also been present at the inquest held by Coroner Hermann, and decided by an unusually intelligent jury, consisting of twelve competent physicians.

As to the result of this inquest and the decision of the jury, we must come to the conclusion that we have now two parties opposed to each other—the one claiming that nitrous oxide is a perfectly safe anæsthetic, and may be given to persons of more or less perfect health, the other desiring to limit the use of this anæsthetic only to cases where there appears to be a necessity, and then only to such persons who according to the state of their health, appear to be able to stand its administration. The first party is composed of interested dentists, while the second party contains a large majority of our scientifically educated physicians, or Practice *versus* Science.

Before I speak on the administration of the gas and its effect on the system, I must state first how the gas is manufactured and the chances of the patient to inhale a gas which is perfectly free from nitric oxide.

The gas is generally prepared from nitrate of ammonia by mere heating. Theoretically the gas is decomposed into water and nitrous oxide. Practically, however, the process is not so simple a one. If the nitrate of ammonia is impure —if it, for instance, contains nitrate of potash, which is an occurrence which not unfrequently takes place—the nitrate of ammonia being prepared from sulphate of ammonia and nitrate of potash—this impurity alone will cause the formation of nitric oxide. But even if it is pure, the formation of nitric acid cannot be prevented. The nitrous oxide acts at a temperature slightly above the temperature when it is formed, reducing the nitrate of ammonia, and forming nitric oxide and nitrate of ammonia, which at once becomes decomposed, forming nitrogen and water. Insertion of a thermometer cannot prevent this accident, for the temperature will not rise unless the pressure is greatly increased inside of the apparatus, just as we are not able to distil water at a temperature of above 100° C. unless we increase the inside pressure of the distilling apparatus. Insertion of a thermometer I therefore deem useless. The bottom of any apparatus, glass or iron, must always be warmer than the mixture, for through it we must conduct the heat which is consumed during the process of preparing the gas. If anything therefore shall be done regarding the heating, it can only be effected by inserting the retorts in metalic or paraffine baths for the purpose of equalizing the affluent of heat. If this is not done the more or less rapid development of the gas will guide the manufacturer sufficiently.

As far as the apparatus is concerned which is used by our New York dentists, it is a fact, that the gas which leaves the retort is always more or less mixed with nitric oxide.—The gas therefore wants purification, which is in all cases done only partially. The better apparatus of this kind has four wash bottles, the first and fourth containing water, the others sol. of potash and diluted sulphuric acid. These two latter substances will free the gas from acids and ammonia. They do not act on nitric oxide, and allow this substance, therefore, to enter the gasometer. If the gas is to be used at once, another substance, either solution of protosulphate of iron or concentrated sulphuric acid, should be employed to absorb any nitric oxide contained in the gas.

It is self understood that the solutions used for the washing of the gas must be frequently renewed, but it is a sad fact that wash bottles supposed to contain free potash have an acid reaction.

From this it would seem as if the public was at all times in imminent danger of being suffocated with nitric oxide, while they suppose they inhale the pure gas. This is, however, rarely or never the case. Nitric oxide is rapidly absorbed by water containing air. If, therefore, the gas enters the large gasometer in an impure condition, the process of absorption at once commences, the construction of the gasometer being always so as to admit the air to the water, which separates the gas from the outer air.

If the gas, however, shall be condensed, a process which lately has been recommended, the gas must be thoroughly purified before it is pumped into the receivers, as then this purification process by water does not occur.

This process of purification is, however, merely accidental, and I have actually met no manufacturer who was aware of this fact; they were all perfectly satisfied that every impurity was taken from the gas by their method of manufacturing; that is by purifying with potash and sulphuric acids.

So much about the manufacturing of the gas. It suffices now to examine the action of the gas on the system, and the process of applying it.

It was formerly supposed, that the nitrous oxide gas acted by means of the comparatively large quantity of oxygen which it contains when compared with our atmospheric air. Our old hand-books on chemistry brought forward theories of this discription, and actually only since the use of nitrous oxide has become common, can we say that nitrous oxide in its influence on the human system has more thoroughly been studied.

These examinations, however, have proved that nitrous oxide does not part with its oxygen in the human system.

It is merely dissolved in the blood, and from the want of chemical affinity under these circumstances allows all the other processes of oxidation, etc., to go on.

One hundred parts of blood of the temperature of the human body are able to absorb sixty volumes of this gas, and yet the spectroscope is not able to show the least changes in the blood. (Dr. Hermann.)

From this it is evident that it is perfectly absurd not only to adhere to the old theory, which is a mere theory and not in the least founded on fact as our new theory is, but more so to recommend the nitrous oxide as an antidote in cases of asphyxia produced by chloroform, from the fact that it contains so much oxygen, as was done lately by a New York dentist, who, to give his words more weight, used the name of Prof. Doremus as the originator of this new theory. I am not informed, however, whether he has been authorized by this gentleman to make such a statement.

Nitrous oxide therefore is supposed to produce its anæsthetic effect by preventing the oxygenation of the nervous centres, and although it contains a large quantity of oxygen, it does not when respired part with any of it; it is expired in the same form as it is inspired.

It is very readily absorbed by the blood; it neither enters into combination with it, nor produces changes in nor suffers changes from the blood.

At the same time the gas allows, when absorbed by the blood, the oxygenation as long as there is any free oxygen present in the blood. It also does not cause any oxygen to leave the blood unused, as the oxygen, what we call free oxygen in the blood, is actually in some form of chemical combination with the blood corpuscles, while the nitrous oxide is merely dissolved in the plasma.

It also allows the carbonic acid to escape from the blood. For this reason it has been found necessary to use such mouth-pieces for the inhalation of the gas as will allow the expired gas to escape, and so prevent the adulteration of the gas with carbonic acid. There have also been proposed other apparatus which are designed to purify the exhaled gas from the carbonic acid by absorption by hydrate of lime.

The first apparatus, with exhale valve, causes a large waste of nitrous oxide, and therefore is by most of our dentists not liked, especially as their bags never contain more than about four gallons of nitrous oxide, a quantity which represents about the average of gas which is used for a single operation.

Dentists who draw, therefore, their supply from the gasometer are the only ones who will use a double-valve mouth-piece with safety, while the others with their small bags would often suffer from interruption of the operation. They use, therefore, single mouth pieces, which will not allow the escape of the exhaled carbonic acid, but compels it to mix with gas not yet inhaled.

The operation of bringing a person completely under the influence of the gas takes about one minute, and during this time about 400 cc. of carbonic acid are expired; these 400 cc. mix, therefore, with about 4 gallons of the gas. The gas, therefore, at the end of the operation, contains about 2½ per cent. of carbonic acid gas, which is certainly a large quantity.

Pettenkofer, in his well-known treatise on respiration, says: "It is not only the heat, nor the degree of saturation of moisture, nor the carbonic acid, nor the wants of oxygen, which makes the air of a room filled with men disagreeable and unbearable for our nerves, and which produces the dull feeling in our head, for such an air becomes repulsive even before it contains 1 per cent. of carbonic acid," etc. And he continues: "It is very probable that some of the organic vapors formed during the process of respiration and perspiration have got but a small tension, and that the air therefore soon becomes saturated, and is not then able to extract more of them from the organism. The retention and accumulation of such vapors in our body, so small as their quantity may be, can certainly act just as well on our nervous system as they act on our nasal organ after they have been liberated by exhalation."

An air which contains but ½ per cent. of carbonic acid, provided that this carbonic acid is produced by exhalation, is unbearable for most persons. Besides that, I must mention that Pettenkofer guards himself by saying that not actually the quantity of carbonic acid makes the air unbearable, but that when he determines the carbonic acid he does it merely to determine the degree of adulteration of the air; he therefore does not consider the carbonic acid the actual poison.

But if, now, an air adulterated with carbonic acid, only to ½ per cent. produces bad effects, how much more an air or gas which contains five times this quantity.

Double-valve mouth-pieces for the inhalation of the gas I therefore consider as absolutely necessary.

The symptoms which are produced by nitrous oxide are about as follows:

From 20 to 30 seconds after the inhalation commences slight lividity of the face is noticed, which gradually increases. After about 50 or 60 seconds the lividity becomes well marked. The amount of lividity present is in all cases very great, and gives to the patient a very unpleasant and dangerous looking appearance. The pupils are slightly dilated, and the breathing is then slower and deeper than natural. At this time the patient is already under the influence of the gas, but in most cases the inhalation is yet continued for 10 to 15 seconds, until slightly stentorous breathing is produced, the pulse remaining all the time full and regular. The pupil is at this time very much dilated, and the patients generally are seized with spasms, during which the breathing is suspended for 10 or 15 seconds yet the pulse remains full and steady. Unsteadiness of pulse is a sign of danger, and the application of gas should in such cases at once be discontinued.

As soon as the breathing commences again the lividity of the face soon subsides.

From this history it is evident that the nitrous oxide produces cessation of the functions of the brain proper, before those of the medulla oblongata; of the medulla oblongata before those of the ganglia of the heart. Hence we have loss of consciousness before failure of the respiration, and this before cessation of the heart.

During the period of the spasms the arterial pressure is greatly increased, and in cases of diseased arteries, therefore, there is danger of rupture of one or more of these arteries.

Authors who have written on this subject, as, for instance, Dr. Charles Squarey, of London, state that in the administration the finger must be kept constantly on the pulse, the breathing carefully watched, and the pupil occasionally examined, especially as the patient is getting under the influence of the gas.

I have, as often as I have seen the gas administered, never seen the operators pay the least attention to the pulse; they all seem to rely merely on the symptoms of breathing.

From the fact that there is suspension of breathing in most cases and that at the same time the arterial pressure is greatly increased, we see that the inhalation of nitrous oxide is not without danger. There are already a number of cases on record in which the nitrous oxide produced death in shorter or longer periods after administration. This number, however, is small, and only includes those cases where death shortly followed the application.

I believe that we soon would learn more of the action of nitrous oxide on the system if the public were aware of the fact that there is danger in taking nitrous oxide. I believe that numerous complaints could be traced back to the day when this most powerful anæsthetic had been given to them. But from the fact that unprincipled men try constantly to impress the public to the contrary, they will always look for other cause, and never suspect the harmless *laughing gas.*—*Druggists' Circular.*

